# The cellular immune system in myelomagenesis: NK cells and T cells in the development of MM and their uses in immunotherapies

**DOI:** 10.1038/bcj.2015.32

**Published:** 2015-04-17

**Authors:** T Dosani, M Carlsten, I Maric, O Landgren

**Affiliations:** 1 Lymphoid Malignancies Branch, Center for Cancer Research, National Cancer Institute, National Institutes of Health, Bethesda, MD, USA; 2 Hematology Branch, National Heart, Lung, and Blood Institute, National Institutes of Health, Bethesda, MD, USA; 3 Hematology Section, Department of Laboratory Medicine, Clinical Center, National Institutes of Health, Bethesda, MD, USA; 4 Myeloma Service, Division of Hematology Oncology, Department of Medicine, Memorial Sloan-Kettering Cancer Center, New York, NY, USA

## Abstract

As vast strides are being made in the management and treatment of multiple myeloma (MM), recent interests are increasingly focusing on understanding the development of the disease. The knowledge that MM develops exclusively from a protracted phase of monoclonal gammopathy of undetermined significance provides an opportunity to study tumor evolution in this process. Although the immune system has been implicated in the development of MM, the scientific literature on the role and status of various immune components in this process is broad and sometimes contradictory. Accordingly, we present a review of cellular immune subsets in myelomagenesis. We summarize the current literature on the quantitative and functional profiles of natural killer cells and T-cells, including conventional T-cells, natural killer T-cells, γδ T-cells and regulatory T-cells, in myelomagenesis. Our goal is to provide an overview of the status and function of these immune cells in both the peripheral blood and the bone marrow during myelomagenesis. This provides a better understanding of the nature of the immune system in tumor evolution, the knowledge of which is especially significant considering that immunotherapies are increasingly being explored in the treatment of both MM and its precursor conditions.

## Introduction

Multiple myeloma (MM) is a malignant neoplasm of plasma cells that arises consistently from asymptomatic precursor conditions, specifically monoclonal gammopathy of undetermined significance (MGUS) and smoldering MM.^1, 2^ The study of myelomagenesis, which is the progression of these precursor conditions to MM, has been an area of interest in the hopes of improving the surveillance and clinical management of these conditions.^3^ Genetic and immune-related factors are considered to have roles in the pathogenesis of both benign monoclonal gammopathies and MM.^4^ Furthermore, two independent groups have developed progression and risk-stratification models for both MGUS and smoldering MM.^5, 6^ Among the parameters used in these models are a skewed free light chain ratio and immunoparesis, which refers to the hypogammaglobulinemia of the uninvolved immunoglobulin.^5, 6^ This suggests that immune dysfunction is an indicator of and may have a role in the progression of precursor disease to MM. Beyond the decrease in humoral immunity, there is also a significant literature that has characterized changes in other components of the immune system in both precursor disease and frank MM.^7, 8^

**Figure 1:**
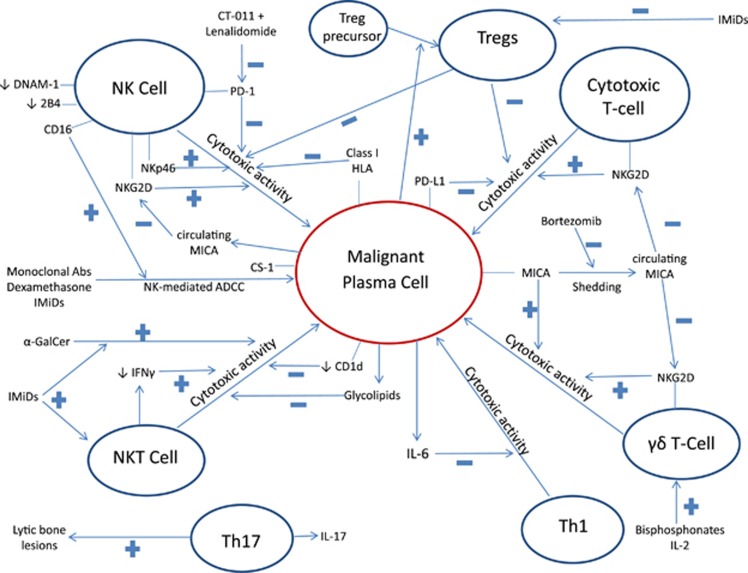
Schematic of functional interactions of NK-cells and T-cells with malignant plasma cells. The functional cytotoxicity of NK-cells against malignant plasma cells is inhibited by malignant plasma cells via the activation of Tregs. MM cells evade cytotoxicity via a lack of HLA Class I loss and the shedding of the surface antigen MICA, which leads to downregulation of the NKG2D activating receptor on NK-cells, cytotoxic T-cells and γδ-T-cells. mAbs and IMiDs rely on NK-cell-mediated ADCC to exert some of their anti-MM effects. Promising targets for NK-mediated immunotherapies against malignant plasma cells include the PD-1/PD-L1 axis and CS1. Circulating MICA is shed by malignant plasma cells upon progression from MGUS to MM and downregulates NKG2D on cytotoxic T-cells, NK-cells and γδ-T-cells. NKT-cells exhibit decreased cytotoxicity from MGUS to MM as evidenced by a loss of IFN-γ production and decreased CD1d-mediated targeting of malignant plasma cells. However, NKT-cells may be stimulated by extrinsic α-GalCer and IMiDs. γδ-T-cells are activated by bisphosphonates and IL-2. Th1 cells are inhibited by IL-6 produced by malignant plasma cells and Th17 cells have a role in the development of bony lytic lesions in MM. Plus and minus signs indicate stimulation or inhibition of pathway demonstrated by arrows, respectively.

Several studies have also discussed the importance of the tumor microenvironment in the development of MM.^9^ Indeed, the term microenvironment is broad and includes a range of various cell types, including immune cells, with varying biological functions (Figure 1). To advance our understanding on this topic, we have conducted an extensive review of the literature on the role of the immune system in myelomagenesis. Here we present an overview of the current knowledge on the status and role of natural killer cells (NK-cells) and T-cells, including conventional T-cells, natural killer T-cells (NKT-cells), γδ-T-cells and regulatory T-cells (Tregs), in myelomagenesis. We focus on these subsets due to their normally cytotoxic activities against tumor cells and their emerging potential in immunotherapies. We emphasize the quantitative (Table 1) and functional (Table 2) profiles of these immune cells in both the peripheral blood (PB) and the bone marrow (BM), with the understanding that interactions between the immune system and tumor cells are significant and distinct in both environments.^9^

This review thus aims to expand our insights on the immune system in myelomagenesis, which is of significance in the development of both immunotherapies and immune biomarkers. Immune biomarkers may be of special relevance in predicting the risk of progression of precursor conditions to MM and would thus be useful in allowing more tailored clinical monitoring and treatment of these patients.^3^ Immunotherapeutic strategies for the treatment of MM continue to show promise in early clinical trials, although they remain to be validated in larger series.^7^ A better understanding of these immune subsets in myelomagenesis will help navigate these new territories.

## Natural killer cells

NK-cells form a distinct subset of the cellular immune system that arises from lymphoid progenitors and is involved in the defense against virally infected and tumor-transformed cells.^10^ NK-cells were first identified owing to their ability to target tumor cells without a need for priming, which stands in contrast to T-cells.^10^ In humans, NK-cells are broadly defined as CD3(−)CD56(+) lymphocytes but express varying levels of other surface receptors that dictate their cytotoxic and immunoregulatory functions.^10^ The common denominator for NK-cell cytotoxicity in humans is the lack of human leukocyte antigen (HLA) expression on the target cell.^10^ Interestingly, in contrast to many tumor types, MM cells often do not show loss of HLA class I, suggesting a mechanism by which they may evade targeting by NK-cells.^11^ Another important pathway that regulates NK-cell cytotoxicity is signaling via the FcyRIIIa receptor (CD16), which mediates antibody-dependent cellular cytotoxicity (ADCC).^10^ However, as we will discuss, NK-cells from MM patients are often dysfunctional compared with NK-cells from healthy individuals.^12^

An initial look at studies of NK-cell counts in the PB of MM patients shows discordant findings, with some showing an increase in NK-cells,^13, 14, 15, 16, 17, 18^ while others reporting no changes^19, 20, 21, 22, 23^ or even a decrease^24, 25^ compared with controls. Similarly conflicting findings are reported in MGUS patients.^15, 17, 19, 21, 25, 26^ A closer look at these PB NK-cell studies reveals differences in the methodologies employed by them, which may explain the variable findings. Most of those studies were not matched by age or sex, both of which may confound the findings due to the known variable NK-cell distributions between age groups and genders.^25^ Moreover, some studies included patients who had previously been treated with chemotherapeutic agents, which may also alter immune cell composition.^26^ Upon filtering out studies with these potential confounders, we found that untreated MM patients in earlier stages of disease generally showed either an increase or no changes in PB NK-cell numbers,^14, 21, 26^ while untreated MM patients in more advanced stages of disease sometimes showed a decrease in PB NK-cell numbers.^25^ MGUS patients also showed no changes in NK-cell numbers, suggesting that changes in PB NK-cell numbers are seen later in the course of disease.^21, 25, 26^ Although these studies hint at the levels of circulating NK-cells in myelomagenesis, future approaches would benefit from the utilization of age- and sex-matched controls and additional stratification of untreated MM patients based on stage of the disease.

The immunophenotypes used in defining NK-cells also varied between these quantitative studies. Although most employed classical NK-cell markers such as CD56 and CD16, they differed in their use of either a single marker or a combination of more than one marker. It may be of value in future studies to delineate NK-cell subsets and their associated findings, particularly because the subsets vary considerably in their functions.^27^ For example, CD56(bright) NK-cells are considered to be the major cytokine-producing subset and are implicated in immune regulation, while the CD56(dim)CD16(+) subsets tend to be more involved in cytotoxic activities.^27^ Interestingly, studies in other cancers have suggested that quantitative levels of certain circulating NK-cell subsets may be useful as clinically predictive biomarkers.^28^ Considering that NK-cell subsets may reflect host immune status, which has been implicated to have a role in the development of MM, they are of interest as markers of immune function in this context. Indeed, MM patients possess decreased levels of CD16(+)subsets,^29^ which are involved in cytotoxicity. Moreover, MM patients with lower NK-cell cytotoxicity show worse disease-free survival than those with higher NK-cell cytotoxicity.^30^ These findings of altered NK-cells and their potential prognostic significance support the exploration of NK-cell subsets as immune biomarkers for MM or its precursor conditions.

Functional studies suggest that, although NK-cells remain functional in MGUS patients, they may display functional changes in MM and lose cytotoxicity particularly in advanced MM.^15, 19, 29, 30^ NK-cell function is often reflected in changes in the NK-cell receptor repertoire, which includes the natural cytotoxicity receptors (NCRs) and NKG2D. Although the expression of NCRs and NKG2D was not found to be different in PB NK-cells from MM patients compared with healthy volunteers,^29^ both NKG2D and NCRs such as NKp30 have been found to be decreased in expression on BM NK-cells.^31^ The co-activation receptor 2B4 has also been found to be reduced in expression in both PB and BM NK-cells.^29, 31^ Other studies in PB NK-cells have suggested that NK-cell cytotoxicity against MM cells is dependent on DNAX accessory molecule 1 (DNAM-1).^32^ However, DNAM-1 has been found to be reduced on NK-cell subsets in MM patients, which may contribute to the decrease in NK-cell functionality observed in MM.^32^ These studies also help highlight that the functional and quantitative status of NK-cells may be distinct between the PB and BM microenvironments. The few published studies on BM NK-cells in MGUS and MM suggest an increased proportion of NK-cells in the BM of patients with these conditions compared with controls.^14, 33^ Functionally, one study of NK-cells from the BM of MM patients showed that they demonstrated strong activity against myeloma cell lines but not against autologous and allogeneic fresh myeloma cells.^34^ The BM microenvironment *in vivo* may thus contribute to the resistance of MM cells to NK-cell-mediated killing.

Although there is an association between advanced disease status and a reduced capacity of NK-cells to mount a proper immune response, it is unclear as to whether disease stage is a consequence of dysfunctional NK-cells or vice versa. Our review of the literature supports sequential studies of the functionality of both NK-cells and the resistance of tumor cells from MGUS to MM in order to better elucidate the order of events over myelomagenesis that leads to both findings. Indeed, MM cells in advanced disease also develop a resistance to NK-cell killing. For example, it has been shown that MM cells are resistant to healthy donor NK-cell-mediated killing in advanced disease^11, 35^ but not in early disease.^11, 36^ Furthermore, a number of pathways in the BM microenvironment milieu inhibit the cytotoxicity of NK-cells, as well as other cytotoxic immune cells (Figure 1). For example, NK-cells from MM patients have been found to have a *de novo* expression of programmed death protein 1 (PD-1), which is involved in preventing host immune response to tumor cells via the PD-1/PD-L1 axis^37^—this axis directly undermines host immune control of MM and allows its clinical progression and has recently also emerged as a promising immunotherapeutic target.^37^ The monoclonal PD-1 antibody CT-011 interrupts this pathway and thus enhances the NK-cell versus MM effect, with this effect also being shown to be augmented by lenalidomide.^37^ Functional studies of NK-cells in disease may also thus help uncover novel immunotherapeutic targets.

Studies of NK-cell functionality in disease are also significant considering that the novel monoclonal antibodies (mAbs) being researched for MM therapy, such as elotuzumab, daratumumab, XmAb5592, and anti-CD137 mAbs, rely on NK-cell-mediated ADCC.^38, 39, 40, 41, 42^ Dexamethasone and immunomodulatory drugs (IMiDs) such as lenalidomide and pomalidomide also exert some of their anti-MM effects via the augmentation of NK-cell cytotoxic activity against tumor cells.^42, 43^ On the other hand, pure NK-cell-based immunotherapies have not yet garnered much success in MM,^42^ although some studies have been promising. One study engineered chimeric antigen receptor-expressing NK-cells and found them to target CS1, which is highly expressed on MM cells and also serves as the antigenic target for elotuzumab.^39, 44^ Another study successfully expanded NK-cells from newly diagnosed untreated MM patients *ex vivo* and showed that they specifically target malignant autologous primary MM cells while sparing their non-malignant counterparts.^45^ This suggests that the functional alterations of NK-cells in MM patients with early disease can potentially be overcome, which is significant when considering their use in immunotherapies. Moreover, immunotherapies may be improved with a better understanding of receptor–ligand interactions that govern NK-cell-mediated targeting of MM.

## T-cells

T-cells have long been known to be both quantitatively and functionally altered in MM and to consequently have a role in the immunodeficiency associated with the disease.^8^ In both MM and its precursor conditions, there is a decrease in the PB CD4(+)/CD8(+) T-cell ratio, which is due to both the decrease in absolute and relative numbers of CD4(+) T-cells and an increase in relative numbers of CD8(+) T-cells.^26, 46, 47, 48, 49, 50, 51^ Moreover, the PB CD4(+)/CD8(+) T-cell ratio has been shown to decrease upon progression of the disease, with the decrease in CD4(+) T-cells correlating with advanced disease, increased tumor burden and as an independent sign of poor prognosis.^48, 50, 52^ In contrast, BM findings in MGUS and MM patients show an increase in CD4(+) T-cells and no significant changes in the CD4(+)/CD8(+) T-cell ratio compared with normal.^33^ An increase in T helper type 1 (Th1) cells in the BM was also noted in both MGUS and MM patients and is consistent with findings of an increased Th1/Th2 ratio observed in the PB of MM patients.^33, 51^ Th17 cells, which are a pro-inflammatory subset of T-cells, are also increased in both the PB and BM of MM patients.^53, 54^ Th17 cells are particularly enriched in the BM, where they are implicated in the development of MM lytic bone lesions via their production of interleukin (IL)-17.^53, 54, 55^

There has historically also been a considerable interest in T-cell clonality, which has been suggested to have prognostic implications in MM.^56, 57, 58^ CD4(+) T-cells in MM patients undergo novel oligoclonal expansions and CD8(+) T-cells, which constitute the majority of the T-cell expansions, demonstrating an increased frequency of expansions.^49, 59, 60^ Tumor-specific CD8(+) T-cells demonstrate strong responses and are correlated with both disease burden and clinical outcomes in MM patients.^56, 57^ Interestingly, both PB and BM studies have shown that clonal CD8(+) T-cell expansions are more frequent in precursor disease patients compared with MM patients, suggesting that cytotoxic T-cell dysfunction correlates with the progression of disease.^33, 61^

These observations are corroborated by functional studies that have shown that, while both CD4(+) and CD8(+) T-cells from the BM of patients with MGUS can effect vigorous tumor-specific responses, these actions are not seen with T-cells from the BM of patients with active MM.^62^ A potential explanation for this phenomenon is that an increasing tumor burden from MGUS to MM may result in T-cell exhaustion. Previous studies have in fact suggested that MM is associated with an increase in CD57(+)CD28(−) cytotoxic T-cells—these are believed to represent a mature subset of T-cells that are generated in response to persistent stimulation by tumor-associated antigen in the absence of effective tumor clearance.^59^ Moreover, sustained co-inhibitory signaling, such as that observed in the PD-1/PD-L1 axis, also results in functional exhaustion of T-cells.^63^ However, T-cell dysfunctionality in MM may also be due to T-cell anergy—this is supported by findings that MM cells induce T-cell anergy by presenting tumor-specific antigens without co-receptor expression.^64^

T-cell-mediated cytotoxicity is also contingent on the function of dendritic cells (DCs), which are the central antigen-presenting cells of the immune system. Considering the breadth of knowledge on DC dysfunction in MM and its consequences on cellular immunity, it is important to highlight these findings. In general, both the PB and BM environments in MM patients generally inhibit DC maturation and function via cytokines and other immunologically active substances.^8^ This leads to a decrease in DC numbers—seen in both the myeloid and the plasmacytoid compartments—and DC function, ultimately contributing to the *in vivo* cytotoxicity of T-cells against MM.^8, 26^ Neither the depletion nor the dysfunction in DCs is seen in MGUS patients, which mirrors findings of intact T-cell-mediated cytotoxicity in precursor disease patients.^26, 65, 66^ Interestingly, T-cells from the tumor bed of MM patients may recover their cytolytic responses against autologous tumor cells after *ex vivo* stimulation with DCs, highlighting the importance of the study of DC function in relation to T-cells in MM.^56, 67^

Successful immunotherapy in MM using conventional T-cell subsets thus needs to bypass barriers of T-cell and DC dysfunctions. However, precursor disease patients may be less immunosuppressed in this regard and thus more amenable to immunotherapeutic approaches of treatment. Recent studies have also increasingly focused their attention on other T-cell subsets, such as NKT-cells, γδ-T-cells and Tregs. We thus specifically address the status and role of these subsets in myelomagenesis.

### Natural killer T-cells

NKT-cells are immune cells that may express both T-cell receptors (TCRs) and NK-cell surface markers.^68^ They are, however, best defined as T-cells that mostly recognize antigens in the context of CD1d molecules, which are related to the major histocompatibility complex family that is utilized by most other immune cells.^68^ Within NKT-cells, the most extensively studied subset are the invariant NKT (iNKT) cells, which are defined by their expression of a highly restricted TCR and their potent activation by the α-galactosylceramide (α-GalCer) ligand.^68^ Despite constituting a minute proportion of all PB T-cells, iNKT-cells have an important role in tumor immunosurveillance and are able to induce strong antitumor responses through the release of cytokines, such as interferon-γ (IFN-γ).^69^

Considering their potent antitumor activities, several studies have attempted to identify the role of iNKT-cells in myelomagenesis. Although some studies have shown a progressive decrease in PB iNKT-cells in the evolution from MGUS to MM,^70^ others have failed to do so.^71, 72^ Comparable studies for BM iNKT cells are not available. Regardless, a decreased functionality of both PB and BM iNKT-cells is seen in MM patients, with the progression of the disease correlating with a loss of IFN-γ production by iNKT-cells.^71^ Studies have also shown the loss of CD1d expression by MM cells upon progression of the disease, suggesting a mechanism by which MM cells evade targeting by iNKT-cells.^70, 71, 73, 74^ Tumor cells themselves may also both express and shed glycolipids that contribute to NKT-cell dysfunction in MM.^71^

Changes in the MM immune environment may thus render iNKT-cells less effective in being able to curb myelomagenesis. Studies have thus attempted to exploit the potent agonist activity of α-GalCer for iNKT-cells. An *ex vivo* study using iNKT-cells from MM patients was able to demonstrate strong antitumor responses against α-GalCer-pulsed primary MM cells.^73^ This was corroborated by a clinical study in smoldering MM patients in which α-GalCer-activated iNKT cells demonstrated a synergistic capacity to induce tumor regression in combination with low-dose lenalidomide.^75^ These studies suggest a role for α-GalCer in immunotherapies looking to rescue or potentiate the antitumor activities of iNKT-cells.

### γδ T-cells

γδ-T-cells comprise a small subset of T-cells that are distinguished by their γδ-TCRs and their distinct effector mechanisms, which include both innate and adaptive features.^76^ γδ-T-cells generally reside and work in tissues rather than in the PB.^76^ However, no quantitative differences have been found in γδ-T-cells in either the PB or the BM of MGUS and MM patients compared with healthy controls.^26, 77^ γδ-T-cells hold immunotherapeutic potential due to their abilities to simultaneously act as antigen-presenting cells, localize to target tissues and rapidly expand and effect antigen-specific immune responses against target cells.^76^ In the context of Phase I/II clinical trials, they have shown efficacy in various cancers, with especially strong responses against advanced renal cell carcinoma and prostate cancer.^76^ These trials either harvested and expanded γδ-T-cells *ex vivo* before introducing them in subjects or activated the cells *in vivo* using bisphosphonates and IL-2, both of which are known to be potent γδ-T-cell stimulators.^76^

There has been an interest in translating these findings to immunotherapies for MM, especially considering that bisphosphonates are already widely used in the treatment of MM.^78^ Pamidronate was among the first bisphosphonates to be investigated and was shown to expand γδ-T-cell populations *ex vivo* in an IL-2-dependent manner.^79^ The expanded γδ-T-cells were then shown to be cytotoxic against both MM cell lines and against plasma cells in BM cultures from MM patients.^79^ This study was corroborated *in vivo* by a clinical trial in which the administration of pamidronate and low-dose IL-2 generated an objective tumor response in MM patients with progressive disease.^80^ However, the tumor response was limited to patients who already possessed expanded γδ-T-cell populations before treatment.^80^ Other bisphosphonates such as zoledronic acid have also been found to activate γδ-T-cells and to enhance their cytotoxic activities against plasma cells derived from MM patients.^81, 82^ Another study utilized Phosphostim, a synthetic γ9δ2-T-cell agonist, to expand γδ-T-cells *ex vivo* and showed that the expanded cells had significant cytotoxicity against most MM cell lines and against primary MM cells.^77^

The antimyeloma activities of bisphosphonate-activated γδ-T-cells may be partially explained by their costimulation by major histocompatibility complex class I-related chain molecule A (MICA), which is an antigenic molecule expressed on the surface of plasma cells from both MGUS and MM patients.^83^ MICA is expressed at higher levels on plasma cells from MGUS patients compared with MM patients, and this is believed to be due to its increased shedding into the PB upon progression of MGUS to MM.^35, 83, 84^ MICA serves as a ligand for the NKG2D receptor, which is present on γδ-T-cells, NK-cells and CD8(+) T-cells, and an increase in circulating MICA triggers the downmodulation of NKG2D on these cells.^85^ The shedding of MICA from MM plasma cells may thus be another mechanism by which they evade immune-mediated cytotoxicity.^85^ Interestingly, the proteasome inhibitor bortezomib augments MICA expression in MM cells, which enhances their targeting by immune cells.^84^ MICA may thus be a promising target in future therapies in MM.

Successful utilization of γδ-T-cells in immunotherapy may necessitate using substances to stimulate them *in situ* or to expand them *ex vivo*. For example, one study derived γδ-T-cells from healthy donors, expanded them for a clone that expressed the NCR NKp44 and showed that this clone effectively targets primary MM cells.^86^ Interestingly, this suggests that the cytotoxic mechanisms of γδ-T-cells might be related to those of NK-cells, which provides rationale for using them synergistically in dual immunotherapies. However, the dearth of γδ-T-cells *in vivo* requires that therapies expand their numbers either *in situ* or *ex vivo*. Other obstacles include their pro-inflammatory tendencies and a relatively poor understanding of their immunobiology.^76^

### Regulatory T-cells

As the major suppressors of immune responses, Tregs have essential roles in the functional homeostasis of the immune system. Because of their phenotypic and functional heterogeneity, studies have often used different definitions for Tregs, which have included positivity for CD25, CD127, FoxP3 or combinations thereof.^87^ These divergent definitions have sometimes resulted in conflicting or non-reproducible findings.^87^ Nonetheless, cancer patients generally show an increased and functional pool of PB Tregs, which suggests that Tregs may have a role in the immunosuppressive state associated with carcinogenesis in MM.^88^

Several studies have shown that CD4(+) Tregs are increased and functionally immunosuppressive in the PB of MM patients.^65, 89, 90, 91, 92^ Most of these studies defined them as CD4(+)CD25(+/high)FoxP3(+) immune cells. However, studies that did not use both the CD25 and FoxP3 markers concurrently^93^ or used a different CD25(high)CD127(low) phenotype^26^ to define CD4(+) Tregs failed to show an increase in Tregs in MM patients. This suggests that the increase in Tregs in MM patients most correlates with the FoxP3(+) phenotype. CD4(+)CD25(+)FoxP3(+) Tregs have also found to be increased and functional in the PB of MGUS patients,^89, 90^ suggesting that Treg-mediated immunosuppression may be an early event in myelomagenesis. Studies of Tregs in the BM are relatively few but tend to suggest no changes in the Treg numbers in MM patients.^90, 92^ Finally, an *in vitro* study showed that both primary MM cells and MM cell lines can generate potent CD4(+)CD25(+)FoxP3(+) Tregs from previously CD4(+)CD25(−)FoxP3(−) cells, suggesting an *in vivo* mechanism by which MM cells may induce immunosuppression and thus evade immune surveillance.^94^ In fact, some of the anti-MM effects of lenalidomide and pomalidomide may be due to their ability to overcome this immunosuppression in MM by their inhibition of the proliferation and function of Tregs.^43^

Although the majority of Tregs are CD4(+), a subset of CD8(+) Tregs have also been identified, although difficulties in phenotypically characterizing them have limited their study.^95^ CD8(+) Tregs are also immunosuppressive and help cancer cells escape immune surveillance via their suppression of effector T-cells.^95^ Similar to their CD4(+) counterparts, the levels of CD8(+) Tregs are increased and functional in the PB of MM patients.^92, 96^ Additional Treg subtypes such as CD4(−)CD8(−) double-negative-Tregs have also been isolated and found to be decreased in MGUS and MM patients.^90, 97^ However, the significance of these novel Treg subsets in carcinogenesis remain to be established.

Tregs are often also studied in relation to the pro-inflammatory Th17 cells, and the Treg/Th17 balance is considered to be a marker in immunoregulatory control.^43^ Interestingly, MM cells skew the Treg/Th17 balance to induce an immunosuppressive state.^98^ Furthermore, the immunological profile associated with long-term survival of MM patients includes a recovery of the Treg/Th17 balance, leading to decreased immunosuppression.^99^ These studies provide additional evidence of the immunosuppression associated with the course of myelomagenesis. They also suggest immune cell subsets that could serve as biomarkers of the development or progression of MM.

## Conclusions and future directions

NK-cells show quantitative and functional changes in myelomagenesis and are particularly decreased and dysfunctional in advanced MM. It remains unclear whether the dysfunction in NK-cells is a cause or consequence of increasing disease stage. In order to elucidate the role of NK-cells in myelomagenesis, we propose a sequential study of changes in NK-cell functionality from MGUS to MM, with a particular focus on how they react with malignant PCs in the BM and changes in the expression of surface receptors. In particular, studies suggest that, in addition to NCRs and NKG2D, the 2B4 and DNAM-1 receptors may have significant roles in NK-cell interactions with tumor cells. Future NK-cell studies may also be improved by controlling for confounding factors and by delineating NK-cell subsets, which may have unequal roles in antitumor activities and thus hold potential as predictive immune biomarkers in myelomagenesis. Finally, functional studies may also help uncover novel immunotherapeutic targets, as evidenced by the recent promising findings of the PD-1/PD-L1 axis and CS1. Indeed, NK-cell-mediated cytotoxicity continues to remain a promising target for immunotherapies, and IMiDs and novel mAbs such as elotuzumab, daratumumab and anti-CD137 mAbs exert significant effects via enhancement of NK-cell-mediated cytotoxicity.

T-cells are significantly altered both quantitatively and functionally in myelomagenesis, and their dysfunction closely mirrors the course of the disease. Immunotherapies using T-cells still present many barriers, although precursor disease patients may be more amenable to such approaches. Recent studies have increasingly focused on NKT-cells, γδ-T-cells and Tregs. NKT-cells and γδ-T-cells have potent agonists that can expand and stimulate their populations. α-GalCer has a promising role in NKT-mediated immunotherapies and should be explored for synergistic use with IMiDs. Bisphosphonates and IL-2 can stimulate γδ-T-cells, which may also be considered for synergistic use with NK-cells. There is insufficient data to suggest a clinical role for T-cell subsets as immune biomarkers in the development of MM. However, circulating MICA and levels of Treg subsets, which reflect the degree of host immunosuppression, may be of significance based on cursory studies suggesting changes in their levels upon the development of MM.

Our discussion of the profiles of both NK- and T-cell subsets in myelomagenesis may thus be helpful in the search for immune biomarkers in MM and its precursor conditions. It also provides knowledge of the baseline profile and the degree of host immunosuppression in myelomagenesis, which is essential when developing new immunotherapeutic strategies for MM. Both NK- and T-cell subsets are already known to have integral roles in the mediation and mechanisms of action of currently explored immunotherapies, particularly those of IMiDs and mAbs—including elotuzumab, daratumumab and anti-CD137 mAbs.^38, 39, 40^ The argument for such immunotherapeutic approaches has been supported by recent findings from massively parallel sequencing of tumor samples from MM patients. These showed that there is widespread genetic heterogeneity in MM,^100^ which is in contrast to the relatively limited immunophenotypic variability in MM. In this context, immunophenotypic homogeneity strengthens the argument for the development of immunotherapies that target defined antigens on MM cells. Future studies are necessary in order to test this hypothesis.

## Figures and Tables

**Table 1:** Quantitative changes of NK- and T-cells in myelomagenesis

	*Peripheral blood*		*Bone marrow*	
	*MGUS*	*MM*	*MGUS*	*MM*
*NK-cells*				
Total	↔^21, 25, 26^	↔ or ↑ in early disease;^15, 22, 27^ ↓ in late disease^25^	↑^33^	↑^14, 33^
				
*T-cells*				
CD4(+)	↔^26, 51^ or ↓^25, 46^	↓^46, 47, 48, 49, 51^		↑^33^
CD8(+)	↔^25, 46, 51^ or ↑^26^	↑^26, 47, 49, 60^		
CD4(+)/CD8(+)	↔^46, 51^ or ↓^25, 26^	↓^26, 47, 48, 50, 51^		↔^33^
Th1			↑^33^	↑^33^
Th1/Th2		↑^51^		
Th17		↑^51^		↑^50, 51^
NKT-cells	↔^70, 71^	↔^71, 72^ or ↓^70^		
γδ-T-cells	↔^26^	↔^26, 77^	↔^26^	↔^26, 77^
CD4(+) Tregs	↑^89, 90^	↑^65, 89, 90, 91, 92^		↔^89, 91^
CD8(+) Tregs		↑^91, 95^		
DNTregs	↓^90^	↓^90^		
Treg/Th17	↔^98^	↑^98^		

Abbreviations: DNTreg, double-negative regulatory T-cell; MGUS, monoclonal gammopathy of undetermined significance; MM, multiple myeloma; NK, natural killer; Th, T helper. ↑, ↓ and ↔ indicate increase, decrease or no changes, respectively, in absolute or relative numbers of corresponding immune cell subsets in the peripheral blood or bone marrow of MGUS or MM patients compared with normal healthy adults.

Blank cells indicate unavailable or insufficient data for the corresponding immune cell subsets upon review of the current literature.

**Table 2:** Functional changes in NK- and T-cells in myelomagenesis

	*MGUS*	*MM*	
*NK-cells*			
	↔	↓	Decreased capacity for ADCC, especially in advanced disease^15, 19, 29, 30^
			Decreased expression of 2B4 and DNAM-1 in MM but without changes in NCRs^29, 31, 32^
			*De novo* expression of PD-1 on MM NK-cells, inhibiting host immune response to tumor cells^37^
			
*T-cells*			
CD4(+) and CD8(+)	↓	↓↓	Loss of tumor-specific responses of both CD4(+) and CD8(+) T-cells in the BM^62^
			Decreased cytotoxic T-cell expansions^33, 61^
			Decreased cytotoxic T-cell-mediated cytotoxicity due to increased DC dysfunction^26, 65, 66^
Th1		↓	Tumor secretion of IL-6 inhibits Th1 function^8^
Th17		↑	Enriched in the BM; involved in the development of lytic bone lesions^53, 54, 55^
iNKT-cells	↓	↓↓	Loss of IFN-γ-mediated antitumor responses of iNKT-cells^71^
			Tumor-secreted glycolipids and decreased CD1d expression on tumor cells contributes to iNKT-cell dysfunction^70, 71, 73, 74^
γδ-T-cells		↓	Downmodulation of NKG2D by circulating MICA shed from tumor cells^35, 83, 84^
			Retain potential for functional stimulation by bisphosphonates and IL-2^79, 80, 81, 82^
Tregs	↑	↑	Tumor cells stimulate the production of functional CD4(+) Tregs^94^

Abbreviations: ADCC, antibody-dependent cellular cytotoxicity; BM, bone marrow; DNAM-1, DNAX accessory molecule 1; IFN, interferon; IL, interleukin; iNKT, invariant natural killer T-cell; MGUS, monoclonal gammopathy of undetermined significance; MICA, major histocompatibility complex class 1-related chain A; MM, multiple myeloma; NK, natural killer; PD-1, programmed death protein 1; Th, T helper; Treg, regulatory T-cell. ↑, ↓ and ↔ indicate increased, decreased and no or minimal changes, respectively, in functionality of corresponding immune cell subsets in MGUS or MM compared with normal.

↓↓ indicates decreased functionality of corresponding immune cell subsets in MM compared with both MGUS and normal.

Blank cells indicate unavailable or insufficient data for the corresponding immune cell subsets upon review of the current literature.
